# Gut microbiome dysbiosis is associated with host genetics in the Norwegian Lundehund

**DOI:** 10.3389/fmicb.2023.1209158

**Published:** 2023-06-19

**Authors:** Claudia Melis, Anna Maria Billing, Per-Arvid Wold, William Basil Ludington

**Affiliations:** ^1^Department of Nature, Environment and Health, Queen Maud University College, Trondheim, Norway; ^2^Department of Embryology, Carnegie Institution for Science, Baltimore, MD, United States; ^3^Department of Biology, Johns Hopkins University, Baltimore, MD, United States

**Keywords:** dysbiosis, domestic dogs, genetic diversity, gut microbiome, outcrossing, *Streptococcus equinus-infantarius-lutetiensis*

## Abstract

A group of diseases have been shown to correlate with a phenomenon called microbiome dysbiosis, where the bacterial species composition of the gut becomes abnormal. The gut microbiome of an animal is influenced by many factors including diet, exposures to bacteria during post-gestational growth, lifestyle, and disease status. Studies also show that host genetics can affect microbiome composition. We sought to test whether host genetic background is associated with gut microbiome composition in the Norwegian Lundehund dog, a highly inbred breed with an effective population size of 13 individuals. The Lundehund has a high rate of a protein-losing enteropathy in the small intestine that is often reported as Lundehund syndrome, which negatively affects longevity and life-quality. An outcrossing project with the Buhund, Norrbottenspets, and Icelandic sheepdog was recently established to reintroduce genetic diversity to the Lundehund and improve its health. To assess whether there was an association between host genetic diversity and the microbiome composition, we sampled the fecal microbiomes of 75 dogs of the parental (Lundehund), F1 (Lundehund x Buhund), and F2 (F1 x Lundehund) generations. We found significant variation in microbiome composition from the parental Lundehund generation compared to the outcross progeny. The variation observed in purebred Lundehunds corresponded to dysbiosis as seen by a highly variable microbiome composition with an elevated Firmicutes to Bacteroidetes ratio and an increase in the prevalence of *Streptococcus bovis/Streptococcus equinus* complex, a known pathobiont that can cause several diseases. We tracked several other environmental factors including diet, the presence of a cat in the household, living in a farm and the use of probiotics, but we did not find evidence of an effect of these on microbiome composition and alpha diversity. In conclusion, we found an association between host genetics and gut microbiome composition, which in turn may be associated with the high incidence of Lundehund syndrome in the purebred parental dogs.

## 1. Introduction

The gut microbiome of an animal is influenced by many factors including diet (De Filippo et al., 2010), exposures to bacteria during post-gestational growth (Torrazza and Neu, 2011; Dowling and Levi, 2014), lifestyle (Clemente et al., 2015; Tun et al., 2017), disease status (Deng and Swanson, 2015), and host genetics (Hughes et al., 2020). Studies suggest a genetic effect on the microbiome composition (Blekhman et al., 2015; Weissbrod et al., 2018; Hughes et al., 2020; Bubier et al., 2021), and differences in gut microbiome composition among dog breeds (Hooda et al., 2012; You and Kim, 2021), although the mechanisms through which specific genes modulate microbiome composition is still unclear. Bubier et al. (2021) describes three mechanisms through which genes could control diseases and link to the microbiome: (1) they might cause the disease phenotype, and microbiome is altered as consequence of disease; (2) they might affect gene expression in the host and indirectly alter the microbiome, which causes the disease; (3) they might affect the microbiome directly and cause the disease through the microbiome. Unraveling these mechanisms requires that we have good models where we can consider host genetics, microbiome, disease phenotype and their relationships simultaneously (Bubier et al., 2021).

Several diseases occurring in humans and dogs, such as Inflammatory Bowel Disease (IBD, including Crohn’s disease and ulcerative colitis), are characterized by an imbalance in the gut microbiota, called “dysbiosis,” where an overgrowth of harmful bacteria, a loss of beneficial bacteria or lowered alpha diversity can occur simultaneously (DeGruttola et al., 2016). It is, however, still unclear whether the dysbiosis is a risk factor or a consequence of the disease (DeGruttola et al., 2016). In both dogs and humans, an association between IBD and dysbiosis has been reported, as seen by an increase of Bacteroidetes (Suchodolski et al., 2012). Additionally, Firmicutes have been found to be decreased in dogs with IBD (Minamoto et al., 2015). However, the dysbiosis in dogs and humans differs in some key bacterial groups (Vázquez-Baeza et al., 2016), possibly due to profound morphological and physiological differences and a relatively recent human adaptation to a more carnivorous diet (Price et al., 2012). For example, *Fusobacterium* appears to be associated with IBD and colorectal cancer in humans, but no association has been established in dogs (Vázquez-Baeza et al., 2016). In humans and mice, obesity has been found to be associated with decreased microbial diversity and an increased Firmicutes/Bacteroidetes (F/B) ratio, whereas in dogs this relationship was not confirmed (Chun et al., 2020; You and Kim, 2021).

The Norwegian Lundehund is a small spitz dog breed that was used to fetch nesting Atlantic puffins *Fratercula arctica* on steep cliffs in the coast of northern Norway (Melis et al., 2013). Towards the end of the 19th century, using nets to hunt puffin became more common than using dogs. Thus, the breed lost its economic importance and was confined to the small fisherman’s village of Måstad on the island of Værøy in the Lofoten archipelago. Two bottlenecks, the first caused by an outbreak of canine distemper in the 1940s, and the second caused by the abandonment of the village of Måstad in the 1960s, left only five highly related individuals. Currently, the breed counts more than 1,500 individuals, which all descend from these five dogs. For this reason, the Lundehund has an extremely low level of heterozygosity, which is around 5% as estimated by high-density SNP arrays (Melis et al., 2022), and an effective population size of only 13 individuals (Melis et al., 2013; Kropatsch et al., 2015). The low genetic diversity is associated with low fertility and with high rates of a protein-losing enteropathy localized to the small intestine, often reported as the Lundehund syndrome, but also as intestinal lymphangiectasia and IBD (Berghoff et al., 2007). Chronic atrophic gastritis and gastric neoplasms are also common in dogs with Lundehund syndrome (Kolbjornsen et al., 1994a,b; Qvigstad et al., 2008). Lundehund syndrome is usually treated by administration of immunosuppressant and anti-inflammatory drugs such as prednisone, prednisolone, or azathioprine and with antibiotics to reduce bacterial overgrowth (Berghoff et al., 2007). However, the extremely low genetic diversity also makes the Lundehund a possible genetic model of gut and autoimmune disease. A mortality study estimated that about 40% of deaths before 11 years of age occurs as a consequence of Lundehund syndrome or other gastrointestinal diseases (Norwegian Lundehund Club, 2014). Although a study found an association between Lundehund syndrome and a missense mutation in the gene LEPREL1 (Metzger et al., 2016), the inheritance mechanism of the Lundehund syndrome is not clear, and it is likely that several genes are involved in the development of this illness (Metzger et al., 2016). Very few individuals in the population do not carry the LEPREL1 mutation, making it impossible to use this information to select breeding individuals, without further reducing the already depauperate gene pool.

Because of the extremely low genetic diversity, the associated disease, and low fecundity, the Norwegian Lundehund Club started an outcrossing project in 2014 with three Nordic dog breeds, the Buhund, the Norrbottenspets, and the Icelandic sheepdog. We focus this study on the Buhund outcross, which started earlier and has, so far, produced the most individuals. The outcrossing project started by mating two unrelated females of Buhund with male Lundehunds, in order to avoid problems with gestation and delivery, due to the difference in relative size, the Buhund being about 30% larger than the Lundehund. The first-generation crossings (F1) were then backcrossed to pure Lundehunds, resulting in the second-generation crossings (F2). All dogs included in the project were screened for hip dysplasia and hereditary ocular pathologies before including them in breeding. We previously analyzed the genetic diversity of the parental, F1, and F2 animals from the Lundehund x Buhund outcross and found a restoration of genetic diversity through outcrossing, with F1 animals having highest diversity (Melis et al., 2022). The mean heterozygosity (estimated from 8,184 linkage-disequilibrium-pruned loci) of Lundehund, F1 and F2 dogs was 0.043, 0.272 and 0.153, respectively (Melis et al., 2022). None of the F1 and F2 progeny has, so far, developed Lundehund syndrome.

**Figure 1:**
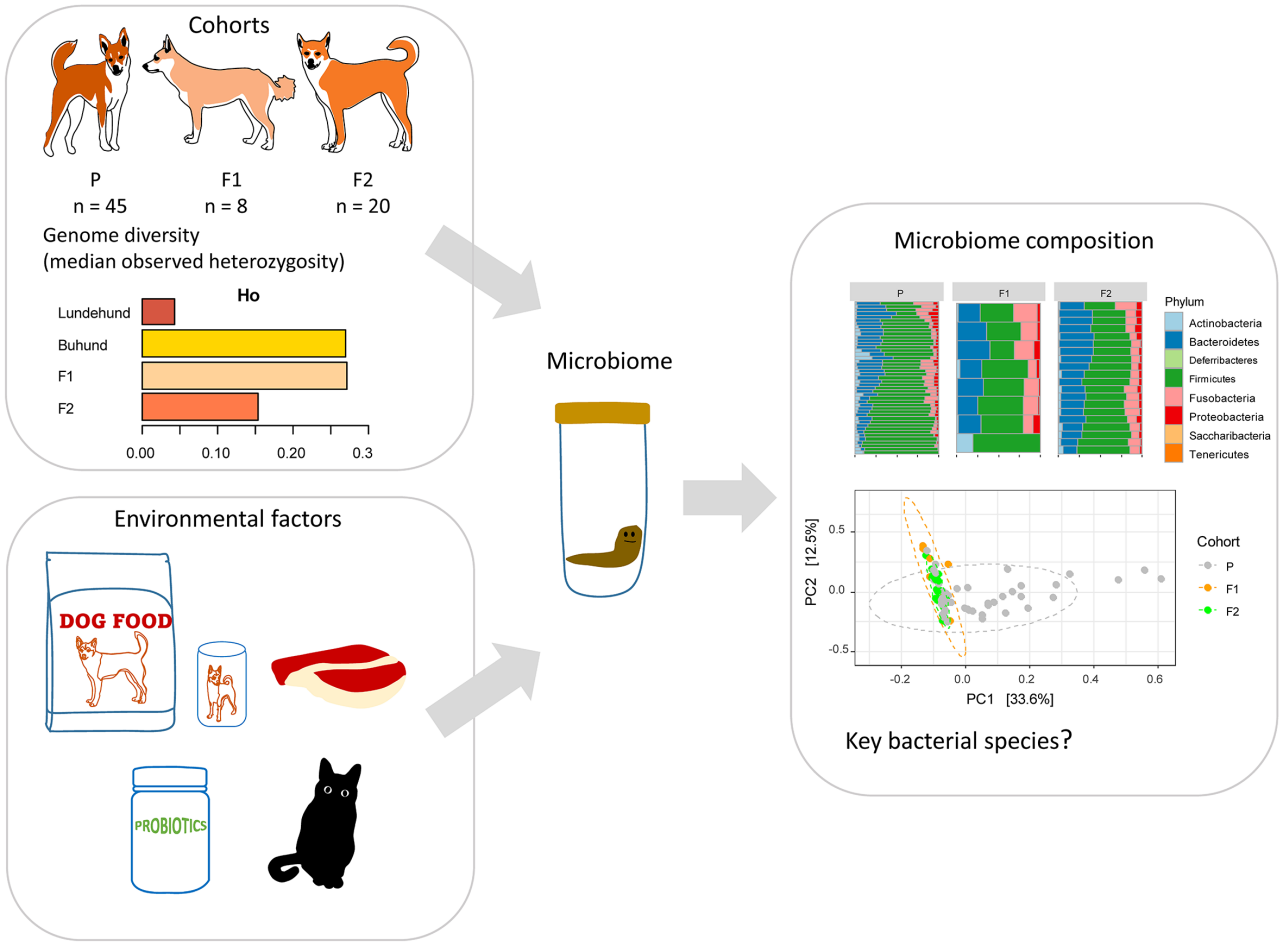
Study design to test whether outcrossing Norwegian Lundehund with Buhund is associated with fecal microbiome composition. The dataset included stool samples from 73 dog individuals: 45 Lundehunds (P), eight first-generation crosses Lundehund × Buhund (F1) and 20 first-generation backcrosses F1 × Lundehund (F2).

To ask whether there might be a microbiome basis for Lundehund syndrome, we sampled stool from parental, F1 and F2 dogs. We analyzed the 16S rRNA gene diversity of the fecal microbiome of purebred Lundehund (P), F1 and F2 individuals (Table 1; Figure 1) with the following aims:

1. Characterize the fecal microbiome composition of Lundehund (P) and first (F1) and second (F2) generation of outcrossings with Buhund.
2. Test whether a range of factors including diet type, presence of a cat in the household, administration of probiotics, and living on a farm correlate with microbiome diversity.
3. Test whether microbiome composition clusters according to cohort (P, F1, F2).
4. Explore whether the ratio between Firmicutes and Bacteroidetes (F/B, an index of dysbiosis) differs between dogs who had Lundehund syndrome and those who did not.

**Table 1:** Phenotypical traits and environmental characteristics of the dogs included in this study.

	Lundehund (P)		F1		F2	
	*n*	%	*n*	%	*n*	%
Gender						
Females	28	56	6	75	10	45
Males	22	44	2	25	12	55
Age (years)						
Mean	5.7	–	5.6	–	3	–
SD	2.7	–	1.06	–	1.34	–
Weight (kgs)						
Mean	7.7	–	9.62	–	9.41	–
SD	1.3	–	2.13	–	2.17	–
Lundehund syndrome history						
Yes	11	22	0	0	0	0
No	39	78	8	100	22	100
Probiotics in the last six months						
Yes	20	40	1	13	1	5
No	30	60	7	88	21	95
Prevalent diet type						
Home made	26	52	2	25	4	18
Industrial dry	16	32	4	50	13	59
Raw	8	16	2	25	5	23
Presence of a cat in the household						
Yes	9	18	3	38	9	41
No	41	82	5	63	13	59
Antibiotics in the last six months						
Yes	5	10	0	0	0	0
No	45	90	8	100	22	100
Home environment						
Farm	3	6	3	38	7	32

Lundehund (P), first-generation crosses Lundehund × Buhund (F1) and first-generation backcrosses F1 × Lundehund (F2).

## 2. Materials and methods

### 2.1. Stool samples collection and analysis

In April to August 2021, we collected stool samples from Lundehund (P, *n* = 50), Lundehund x Buhund crosses (F1, *n* = 8), and F1 × Lundehund crosses (F2, *n* = 22). The lower number of individuals in the F1 cohort is due to the challenge in finding owners of Buhund females that were willing to let their dog being paired with a Lundehund. Owners were instructed in how to collect and handle fresh naturally deposited samples, avoiding contamination. The stool samples were stored at room temperature in Stool Nucleic Acid Collection and Preservation Tubes (Norgen BioTek Corp, Cat. 45,660). In September 2021, the samples were analyzed with the ZymoBIOMICS® Targeted Sequencing Service (Zymo Research, Irvine, CA). The ZymoBIOMICS®-96 MagBead DNA Kit (Zymo Research, Irvine, CA) was used to extract DNA using an automated platform. Bacterial 16S ribosomal RNA gene targeted sequencing was performed using the Quick-16S™ NGS Library Prep Kit (Zymo Research, Irvine, CA). The bacterial 16S rRNA primers amplified the V3-V4 region of the 16S rRNA gene. The final PCR products were quantified with qPCR fluorescence readings and pooled together based on equal molarity. The final pooled library was cleaned with the Select-a-Size DNA Clean & Concentrator™ (Zymo Research, Irvine, CA), then quantified with TapeStation® (Agilent Technologies, Santa Clara, CA) and Qubit® (Thermo Fisher Scientific, Waltham, MA). The ZymoBIOMICS® Microbial Community DNA Standard (Zymo Research, Irvine, CA) was used as a positive control for each targeted library preparation. Negative controls (i.e., blank extraction control, blank library preparation control) were included to assess the level of bioburden carried by the wet-lab process. The final library was sequenced on an Illumina® MiSeq™ with a v3 reagent kit (600 cycles). The sequencing was performed with a 10% PhiX spike-in. Unique amplicon sequences variants (ASVs) were inferred from raw reads using the DADA2 pipeline (Callahan et al., 2016). Potential sequencing errors and chimeric sequences were also removed with the DADA2 pipeline. ASVs that were present in only one sample, i.e., singletons, were examined for each cohort and removed from the dataset for clustering analysis. Taxonomy assignment was performed using Uclust from Qiime v.1.9.1 (Caporaso et al., 2010) with the Zymo Research Database. Any taxa that were not represented at over 1% relative abundance in at least one sample were removed. Five purebred Lundehund dogs older than 9 years and two F2 dogs that were under medication at sampling were also removed from further analyses, in order to obtain a sample more homogenous in age and without the influence of antibiotics. Normalization of the data was performed by calculating the relative abundance for each sample by library scaling.

### 2.2. Microbiome statistical analyses

The microbiome analyses were done with a dataset including 306 bacterial species and 73 individual samples in R version 4.1.3 (R Core team, 2021) with RStudio version 2022.07.2 (RStudio Team, 2022) and with the R packages *Phyloseq* (McMurdie and Holmes, 2013) and *Microbiome* (Lahti et al., 2017). We explored the microbiome composition by plotting the relative abundance of bacterial phyla and of the genera present at >1% relative abundance of which there were 15. These plots were produced with the function *comp_barplot* from the package *microViz* version 0.10.8 (Barnett et al., 2021). The R package *plyr* (Wickham, 2011) was used to calculate richness and diversity of bacteria according to the different categories reported in Table 1. We tested for a statistical difference in relative abundance of all phyla and the subset of 15 most abundant genera with the function *xdc.sevsample* in the Human Microbiome Project (*HMP*) R package version 2.0.1 (La Rosa et al., 2012). This function performs a multivariate test for differences in composition between groups assuming Dirichlet-multinomial distribution by testing for a difference in the mean distribution of each taxon across groups and also account for the overdispersion in the count data (Wilks, 1938). Differences in relative abundances of specific phyla between groups were compared by the R package *Maaslin2* (Mallick et al., 2021) with Benjamini-Hochberg correction to control for false discovery rate. For this analysis, due to the low number of F1 individuals, the F1 and the F2 generations were pooled together and compared to the purebred Lundehund generation (P).

Alpha diversity, as calculated by the Shannon index, was compared across groups according to Table 1. As a measure of beta diversity, a principal component analysis at species level was performed with the R package *Phyloseq* (McMurdie and Holmes, 2013) and the command *ordinate* and “RDA” method (the distance method on the Bray Curtis distance). Comparisons between Shannon indices and F/B ratios were performed by Wilcoxon tests with Bonferroni correction. We also tested whether the samples clustered to a higher degree than expected by sampling variability using permutational multivariate analysis of variance (PERMANOVA). For the permutational analysis the distance method was set to “Euclidean.”

### 2.3. Environmental variables

A questionnaire was also sent to the dog owners, together with the stool sampling tubes, to obtain information about environmental factors such as the typical diet, Lundehund syndrome history, antibiotics and probiotics administration (in the previous 6 months), presence of a cat in the household and whether the dog lived on a farm or in a more urban environment (Table 1). Many of the purebred Lundehunds (40%) used a combination of two to three different types of probiotics, whereas probiotics administration was less common among F1 and F2 dogs.

## 3. Results

### 3.1. Microbiome compositional variation is correlated with dog genotype

To assess whether Lundehund syndrome is associated with gut dysbiosis, we collected stool samples and metadata (Table 1) from 73 dogs, comprising 45 purebred Lundehunds, 8 Lundehund × Buhund F1 animals, and 20 F1 x Lundehund crosses, which are the F2 generation. To examine the microbiome of the Norwegian Lundehund and F1 and F2 outcrossing generation, we assessed the bacterial taxonomic composition of the stool by 16S rRNA gene amplicon library sequencing. After assigning taxonomy to the bacteria in each sample based on ASV and filtering the data to remove singletons and rare taxa, the final microbiome data set included 306 bacteria species and 73 individual samples. Five ASVs, with taxonomic assignments to *Collinsella intestinalis-stercoris*, *Blautia hansenii-producta*, *Lachnoclostridium* sp32341-sp32430, Clostridiales (no species), and *Fusobacterium mortiferum*, were present in all samples. A loss of alpha diversity has also been shown by various studies to correlate with microbiome-associated disease. We detected no significant differences in alpha diversity, as calculated by Shannon index (Appendix S1), as a function of dog generation, Lundehund syndrome history, diet type, administration of antibiotics, administration of probiotics, presence of a cat in the household, or living on a farm versus in a suburban environment (Wilcoxon rank sum test, all *p >* 0.05), although we note that the potential influence of these factors on microbiome composition cannot be ruled out due to the relatively small sample size.

**Figure 2:**
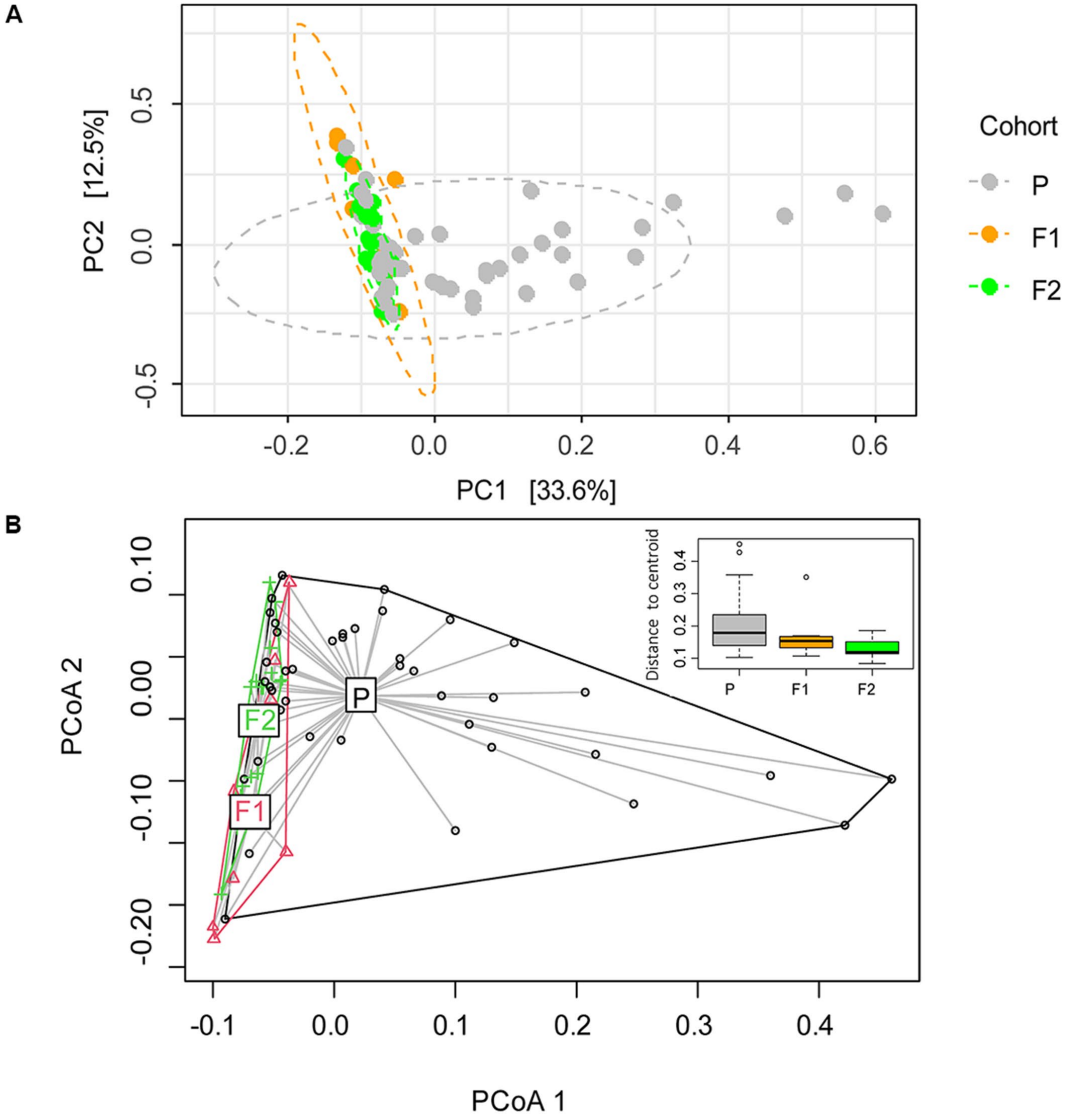
**(A)** Ordination analysis performed with *Phyloseq* on gut microbiome of 73 dog individuals, including 45 Lundehunds (P), eight first-generation crosses Lundehund × Buhund (F1) and 20 first-generation backcrosses F1 × Lundehund (F2). **(B)** Ordination centroids and dispersion measured by Aitchison distance on gut microbiome composition of the same dataset.

To further assess the microbiome compositional variation between dogs (beta diversity), we performed a principal component analysis at the species level (Figure 2). By defining the centroid of variation for each of the dog generations, we found that purebred Lundehunds were clearly differentiated from F1 and F2 dogs based on principal component #1 (PC1), which explains 33% of the variation in the data (Figure 2A). Whereas there was wide variation in PC1 for purebred Lundehunds, the F1 and F2 generations were much more similar to one another (Figure 2A). The distance-based test of homogeneity of multivariate dispersions showed that the samples clustered beyond the expectation from sampling the total variability (*F* = 7.3, df = 2, *p* = 0.002, Figure 2B). We also performed a principal component analysis with a dataset including only purebred Lundehunds and plotted the ordination by Lundehund syndrome history (Appendix S2). The ellipses overlapped almost totally, showing that there is no difference in beta diversity between purebred Lundehunds which had a Lundehund syndrome history and those which did not.

Overall, our results indicate that microbiome composition is associated with the genetic background of the dogs. The majority of the variation occurred in purebred Lundehunds, suggesting that these dogs lack some control mechanism regulating their microbiome composition. We found no evidence that other known factors associated with microbiome disease contribute to the microbiome composition in Lundehunds.

### 3.2. Lundehunds have a microbiome compositional signature at the phylum and the genus levels

**Figure 3:**
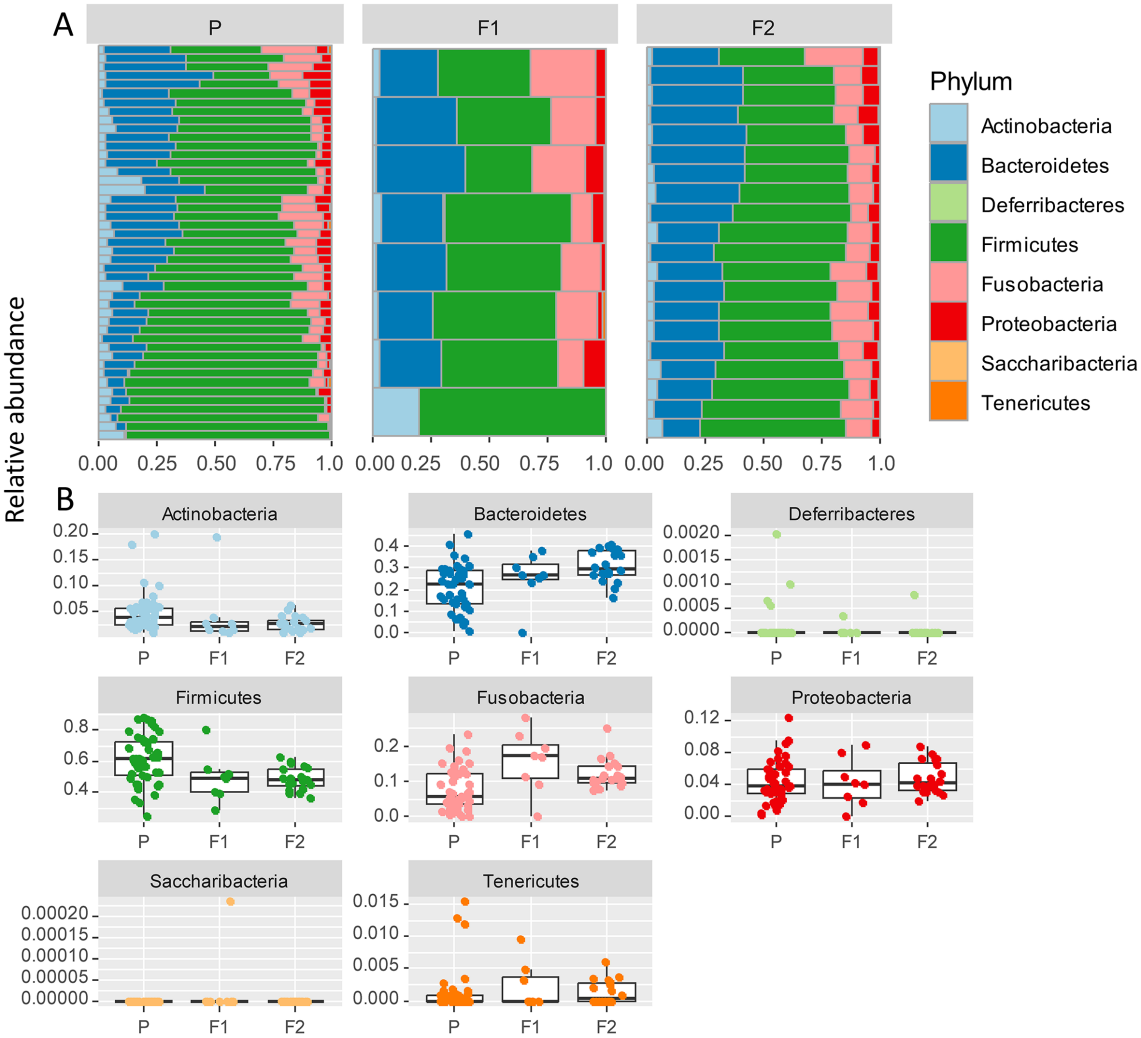
**(A)** Relative abundance of bacterial phyla identified in stool samples from 73 dog individuals, including 45 Lundehunds (P), eight first-generation crosses Lundehund × Buhund (F1) and 20 first-generation backcrosses F1 × Lundehund (F2). **(B)** Box and whiskers plots of the relative abundance of bacterial phyla identified in stool samples from 73 dog individuals. Whiskers represent 1.5 times the interquartile range past the low and high quartiles. Points outside whiskers range are outliers.

The most abundant bacterial phyla across all samples were Firmicutes (57%), Bacteroidetes (23%), and Fusobacteria (10%), followed by Proteobacteria (4%), and Actinobacteria (4%; Figure 3A). To assess which were the compositional differences based on the genetic background of the dogs, we examined the composition of bacteria at the phylum level. We expected that any taxa that are associated with the Lundehund genetic background should be highest in Lundehund, lowest in the F1 generation, and intermediate between Lundehunds and F1 in the F2 backcrosses. Phylum-level differences in relative abundances were evident based on purebred versus outcrossed status of the dogs (Figure 3A).

We plotted the relative abundance of each phylum for each generation of dogs (Figure 3B). The relative abundance at the phylum level was significantly different between the three generations (X _several sample test_ = 53.65, *p* = 6 × 10^−6^). Overall, the F1 and F2 progeny were more similar to each other in their microbiome composition than to the purebred dogs. Due to the low number of F1 dogs, we combined the F1s and F2s and compared the phylum-level microbiome abundances for these with the purebred Lundehunds. The abundances of Actinobacteria and Firmicutes were higher in purebred Lundehunds than in the F1 and F2 progeny (both *p* = 0.007). The relative abundance of Fusobacteria showed the opposite pattern, with a lower abundance in purebreds than in the F1 and an intermediate abundance in the F2 progeny (*p* = 0.04). The F1 and F2 dogs also had lower variance in composition at the phylum level (Figure 3B), consistent with the variation observed by principal component analysis (Figure 2A).

**Figure 4:**
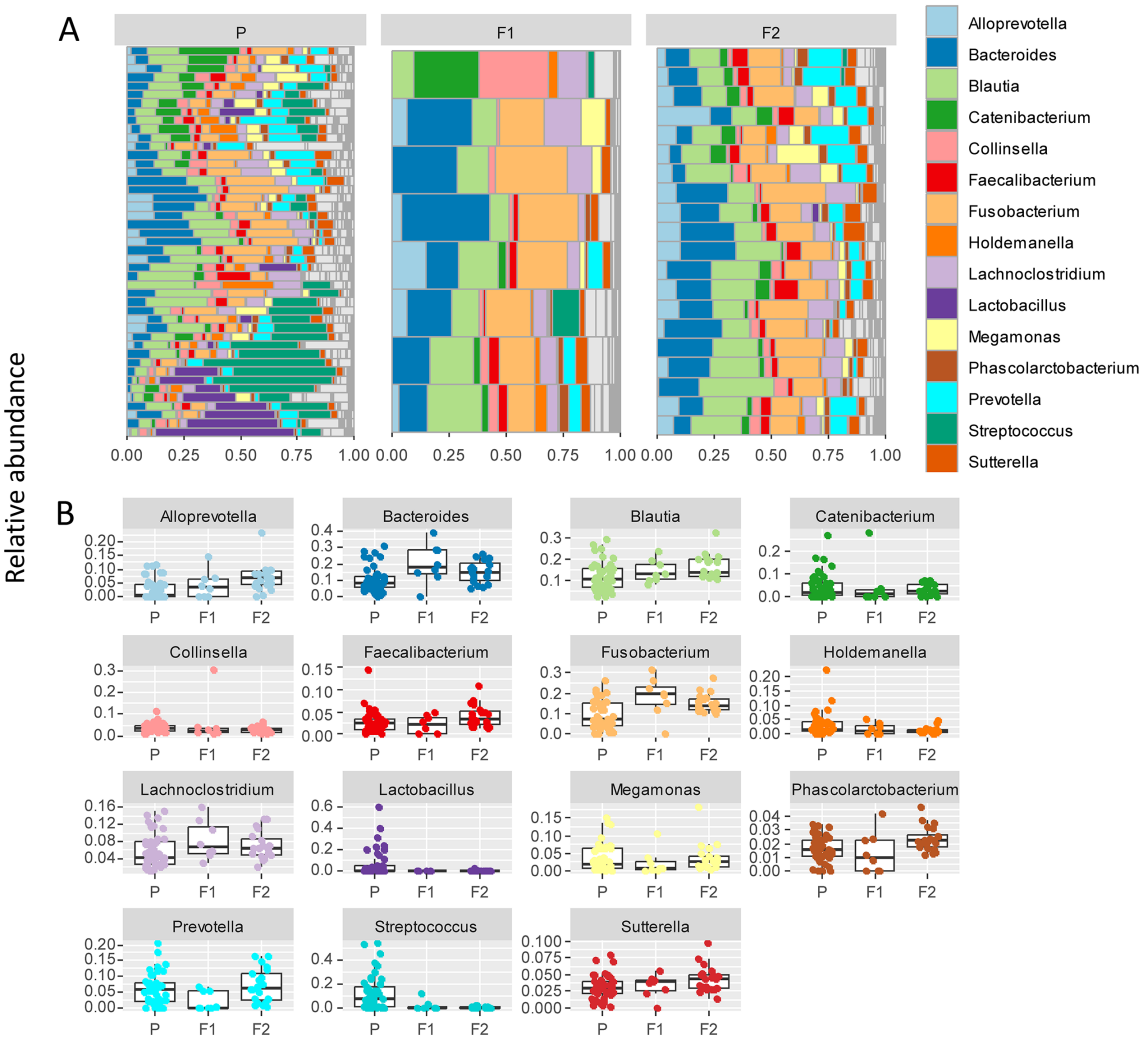
**(A)** Relative abundance of the 10 most abundant bacterial genera identified in stool samples from 73 dog individuals, including 45 Lundehunds (P), eight first-generation crosses Lundehund × Buhund (F1) and 20 first-generation backcrosses F1 × Lundehund (F2). **(B)** Box and whiskers plots of the relative abundance of the 10 most abundant bacterial genera identified in stool samples from 73 dog individuals. Whiskers represent 1.5 times the interquartile range past the low and high quartiles. Points outside whiskers range are outliers.

To further delineate bacterial taxa associated with the different dog genetic backgrounds, we performed the same analyses as in Figure 3 at the genus level on a subset including the genera present at >1% relative abundance across all samples, amounting to 15 highest abundance genera (Figure 4A). These analyses also revealed some differences between cohorts. To examine more in detail the genus-level variation in relative abundance in the purebred Lundehunds versus F1 and F2 progeny, we plotted the relative abundance of the 15 most abundant genera for each generation of dogs (Figure 4B). The relative abundances of genera were overall significantly different between the three cohorts (X _several sample test_ = 110.39, *p* = 4 × 10^−11^). We next made pairwise statistical tests comparing the relative abundance of each genus between purebred and outcrossed dogs (F1 and F2). We detected significant differences for 7 genera by doing pairwise tests, indicating these specific genera are associated with the genetic background of the dogs. *Streptococcus* (*p =*3 × 10^−7^), *Lactobacillus* (*p =* 0.0005), and *Holdemanella* (*p =* 0.01) were at higher abundance in the purebred Lundehunds whereas *Alloprevotella* (*p =* 0.003), *Blautia* (*p =* 0.005), *Lachnoclostridium* (*p =* 0.02) and *Fusobacterium* (*p =* 0.02) were at higher abundance in the outcrossed dogs. Overall, we detected significant associations between specific bacterial taxa and the Lundehund genetic background, indicating a host genetic basis for the gut microbiome compositional differences.

Since changes in the ratio of Firmicutes to Bacteroidetes have been associated with microbiome dysbiosis in numerous studies of microbiome-associated diseases (e.g., Suchodolski et al., 2012; Minamoto et al., 2015; Vázquez-Baeza et al., 2016; Chun et al., 2020; You and Kim, 2021), we computed the Firmicutes to Bacteroidetes ratio for each dog and compared between the generations. We found that purebred Lundehunds have a higher Firmicutes to Bacteroidetes ratio than F1 and F2 progeny (Wilcoxon test, W = 943, *p* = 0.0003). We note that no Lundehund syndrome has been detected to date in any of the F1 or F2 progeny.

### 3.3. The Lundehund microbiome is not indicative of Lundehund syndrome

**Figure 5:**
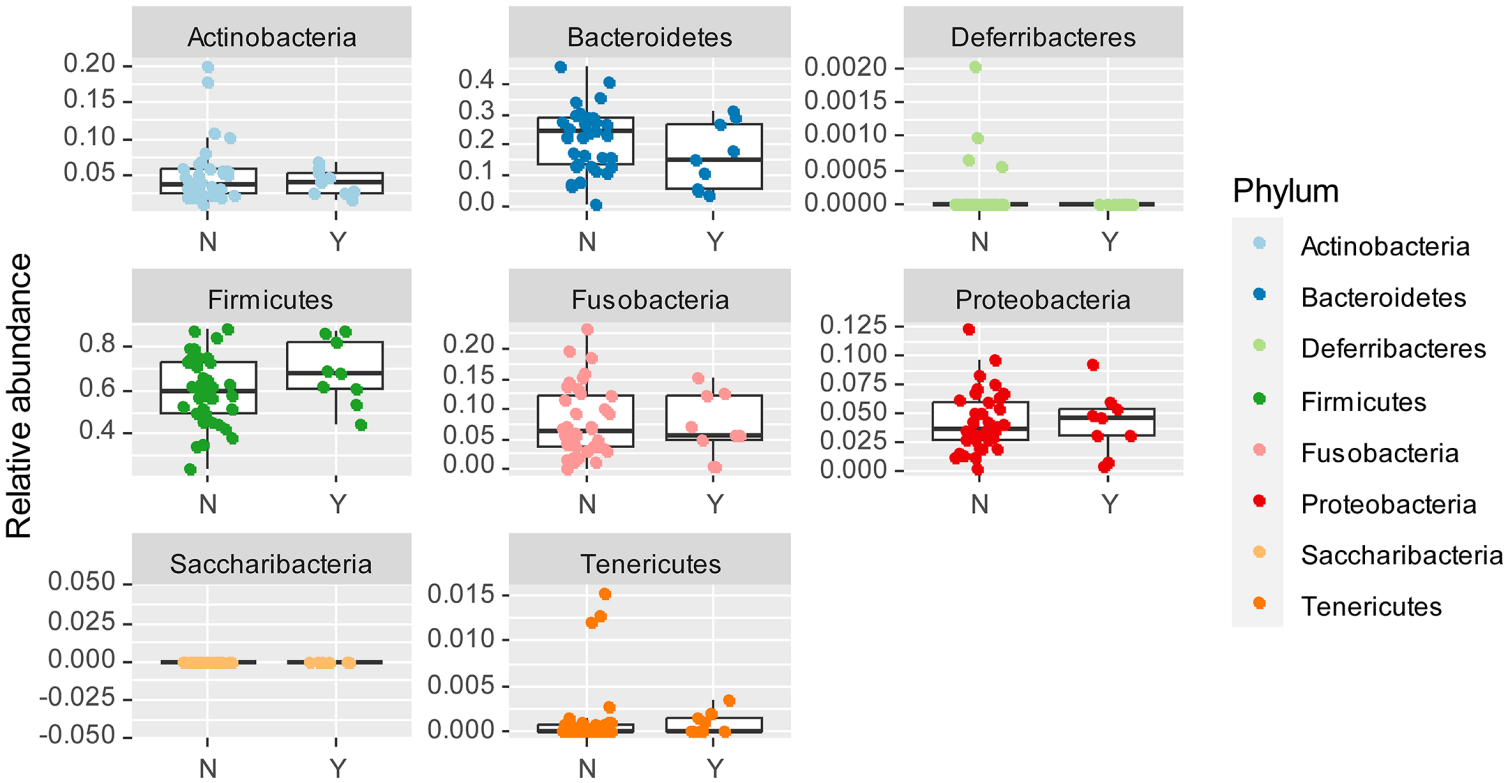
Box and whiskers plots of the relative abundance of bacterial phyla identified in stool samples from dogs which Y = had Lundehund syndrome (LS), and N = which did not have LS. Whiskers represent 1.5 times the interquartile range past the low and high quartiles. Points outside whiskers range are outliers. The data set includes 45 purebred Lundehunds (P).

To examine whether Lundehund syndrome is also associated with microbiome composition, we plotted the relative abundance of each phylum as a function of Lundehund syndrome status (Figure 5) within the purebred Lundehunds. The relative abundance of phyla did not differ significantly between purebred Lundehund who had a diagnosis of Lundehund syndrome at some point in their life versus those who did not (X _several sample test_ = 7.56, *p* = 0.48).

To further examine whether there is a Lundehund syndrome microbiome composition, we calculated the Firmicutes to Bacteroidetes ratio for purebred Lundehunds with and without a history of Lundehund syndrome. The median ratio of Firmicutes to Bacteroidetes was 4.41 (*n* = 9) in dogs who had Lundehund syndrome versus 2.25 (*n* = 36) in dogs who did not. While there is a trend of a higher ratio in dogs with a history of Lundehund syndrome, these medians were not significantly different between the two groups (Wilcoxon test, W = 215, *p* = 0.1).

**Figure 6:**
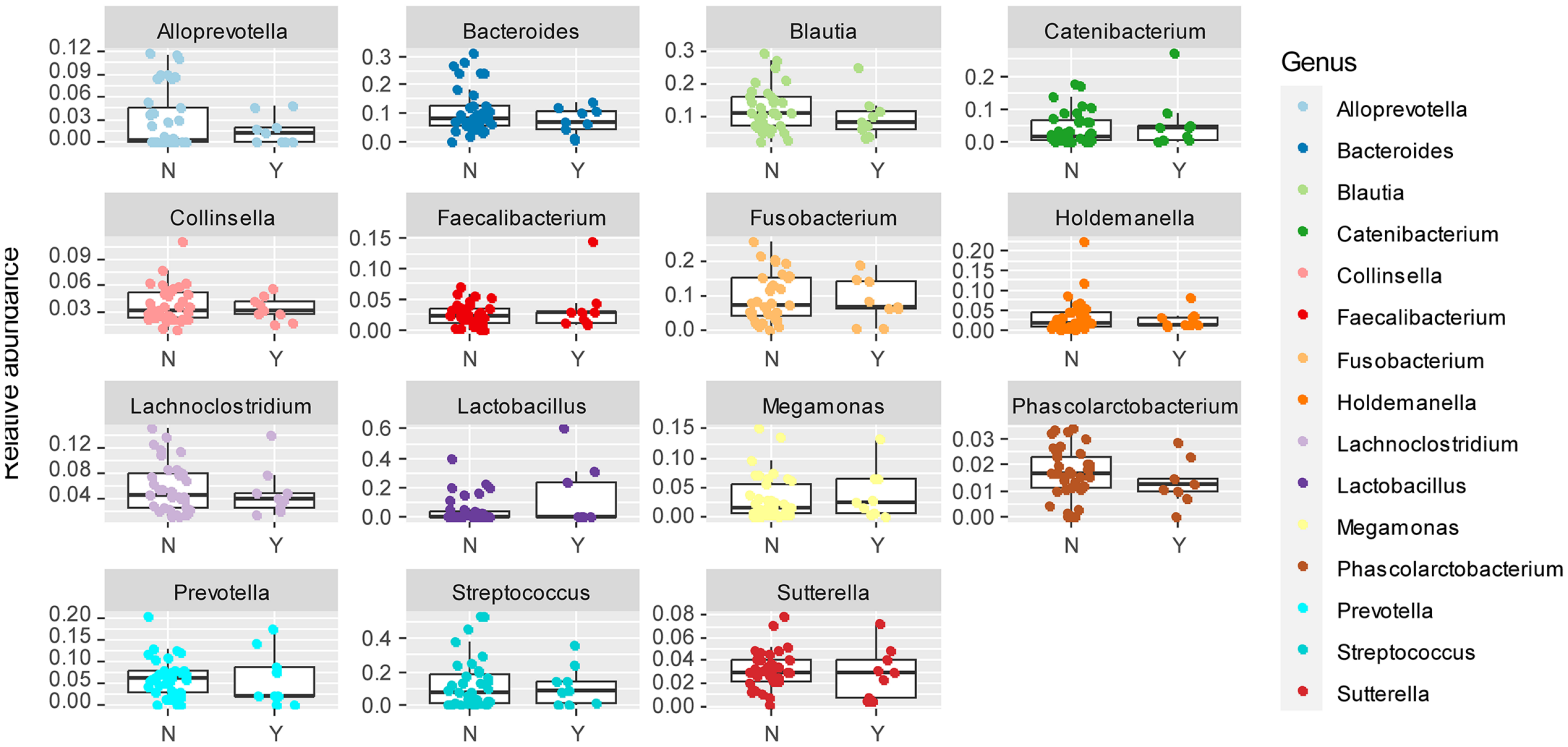
Box and whiskers plots of the relative abundance of the 15 most abundant bacterial genera identified in stool samples from Lundehunds which Y = had Lundehund syndrome (LS), and N = which did not have LS. Whiskers represent 1.5 times the interquartile range past the low and high quartiles. Points outside whiskers range are outliers. The data set includes 45 purebred Lundehunds.

To further delineate the microbiome associated with Lundehund syndrome, we plotted the relative abundance of the 15 most abundant genera as a function of Lundehund syndrome status (Figure 6). Purebred Lundehunds who had a diagnosis of Lundehund syndrome at some point in their life did not differ significantly in the relative abundance at genus level compared to healthy dogs (X _several sample test_ = −10.11, *p* = 1). Overall, we found no compositional differences that were significantly associated with Lundehund syndrome within the purebred dogs, consistent with the genotype of the dogs driving the compositional differences we observed between generations.

### 3.4. *Streptococcus equinus-Infantarius-lutetiensis* is more abundant in purebred Lundehund

In examining the variation in microbiome composition (Figure 2) and the phylum- and genus-level differences in abundances between purebred dogs and the F1 and F2 progeny (Figures 3, 4), we noticed that the F1 and F2 generations appeared similar to one another and had overall more consistent abundances of each bacterial phylum and genus, whereas purebred Lundehunds have much wider variation in microbiome composition.

**Figure 7:**
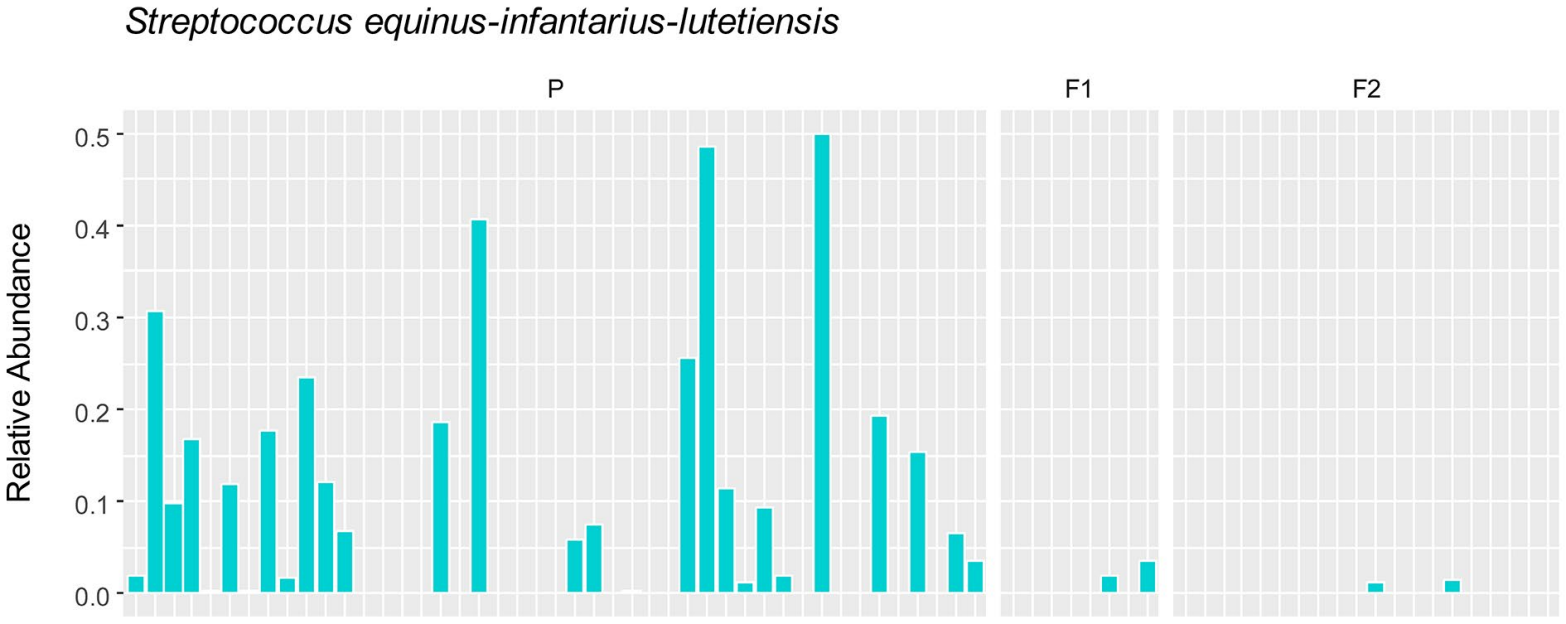
Relative abundance of *S. equinus-infantarius-lutetiensis* in gut microbiome of 73 dog individuals, including 45 Lundehunds (P), eight first-generation crosses Lundehund × Buhund (F1) and 20 first-generation backcrosses F1 × Lundehund (F2).

To assess whether any specific taxa corresponded to the differences in the variation based on principal component analysis, we examined the major contributing taxa to principal component 1, which clearly differentiated the purebred dogs from the F1 and F2 generations (Figure 2). Based on the loadings (Table 2), we found that *S. equinus-infantarius-lutetiensis* was by far the most important species for principal component #1. We next examined the abundance of *S. equinus-infantarius-lutetiensis* in the individual samples and found that this bacterium is much more abundant in purebred Lundehunds and almost absent in F1 and F2 dogs (Figure 7), indicating that, in addition to the dysbiosis signature in the microbiome in the principal component analysis (Figure 2), and the Firmicutes/Bacteroidetes ratio, Lundehunds also have a characteristic species, *S. equinus-infantarius-lutetiensis*, which is associated with the dysbiosis. When examining the relative abundance of *S. equinus-infantarius-lutetiensis* within the pure bred Lundehunds, we did not find any pattern related to Lundehund syndrome (Appendix S3), indicating that the association is not a potential causative agent in accord with Koch’s first postulate on infectious disease (Evans, 1978) which says that the microorganism must be found in the diseased animal, and not found in healthy animals. Rather, *S. equinus-infantarius-lutetiensis* is a species associated with dysbiosis in Lundehunds.

**Table 2:** Most important species dominating the first component of the PCA on microbiome composition of 73 dog individuals, including 45 Lundehund (P), eight first-generation crosses Lundehund × Buhund (F1) and 20 first-generation backcrosses F1 × Lundehund (F2).

Sequence	PCA1	Species
102	0.9304039	*Streptococcus equinus-infantarius-lutetiensis*
34	−0.1612259	*Bacteroides* sp12209
274	−0.1372722	*Fusobacterium mortiferum*
278	−0.1278976	*Fusobacterium* sp37464
53	−0.1040231	*Alloprevotella* sp13496-sp13497
195	−0.0764320	*Clostridiales* sp.
147	−0.0737325	*Lachnoclostridium* sp32341-sp32430
88	0.0693046	*Lactobacillus reuteri-vaginalis*
72	0.0663620	*Lactobacillus* sp.
124	−0.0635586	*Blautia hansenii-producta*

## 4. Discussion

This study examined the microbiome of purebred Lundehund dogs compared with first and second-generation outcrossings with the Buhund. Consistent with previous studies on the dog fecal microbiome, the dominant phyla in all cohorts were Firmicutes, followed by Bacteroidetes and Fusobacteria (e.g., Swanson et al., 2011; You and Kim, 2021).

We sampled privately owned dogs that had a range of diet and probiotic regimes, therefore our dataset included several potentially confounding factors, such as diet, probiotics regime, different living conditions and age classes. Moreover, Lundehund syndrome is an acute life-threatening disease, which requires immediate treatment with anti-inflammatory drugs and antibiotics. For this reason, we could not collect samples from sick dogs before antibiotics administration and we thus compared the fecal microbiome of healthy dogs (which never had a diagnosis for Lundehund syndrome) with that of dogs which had recovered from the illness.

Despite these limitations, we detected a signature of purebred Lundehund status in the microbiome composition when comparing the different generations of dogs. Given the expectation that Lundehund genetic background-associated taxa should be high in purebred dogs, low in F1s, and intermediate in F2, Firmicutes and Actinobacteria appear Lundehund-associated, whereas this was not the case for Fusobacteria and Bacteroidetes. Our results are consistent with several studies which showed that sequences belonging to the phylum Bacteroidetes decreased in dogs with acute diarrhea compared to healthy dogs (Chaban et al., 2012; Guard et al., 2015). Fusobacteria have also been found to be decreased in dogs with clinically active inflammatory bowel disease (IBD; Suchodolski et al., 2012) and are generally associated with a healthy microbiota (Vázquez-Baeza et al., 2016; Pilla and Suchodolski, 2020).

An increased or decreased F/B ratio is considered as a sign of imbalance in the intestine, or dysbiosis. An increased F/B ratio is often observed in humans with obesity (Abenavoli et al., 2019), although there are contradictory results (Chun et al., 2020; Magne et al., 2020; You and Kim, 2021), whereas a decreased F/B ratio is observed in the intestine of humans with IBD (Shen et al., 2018). We observed a significantly increased F/B ratio in purebred Lundehunds. However, contextualizing this result, we might not necessarily expect to be able to compare the relative abundances of functional bacterial groups in humans and dogs, since they have evolved under different pressures, such an omnivorous diet in humans versus a carnivorous diet in dogs (Vázquez-Baeza et al., 2016).

We could not observe any correlation between environmental factors and microbiome alpha diversity as calculated by the Shannon index, but the small size of the dataset and the coexistence of several factors, could make it difficult to disentangle their effects. Despite that, microbiome composition (beta diversity) clustered according to cohort, revealing a signature of the genome in the microbiome. When purebred Lundehunds were compared with F1 and F2 crosses, the variance in microbiomes was larger within the purebred Lundehunds than within F1 and F2 animals. Higher microbiome disparity was evident in purebred Lundehunds when examining the PC1 in the principal component analysis (Figure 2), suggesting the loss of a control mechanism over microbiome composition in Lundehunds.

Interestingly, we found that a single taxon, *S. equinus-infantarius-lutetiensis* was the most important species driving PC1 (explaining 33% of variation in the data). *S. equinus-infantarius-lutetiensis* was much more abundant in the purebred Lundehund, than in the F1 or F2. Several studies suggest an association between gut diseases in humans and bacteria belonging to the *S. bovis* group, which includes *S. equinus-infantarius-lutetiensis*. *S. equinus-infantarius-lutetiensis* was for instance isolated in stool samples of children with diarrhea of unknown origin, suggesting its pathogenic potential (Jin et al., 2013). This bacterium has also been linked to colorectal carcinogenesis in humans, since it could be found at higher rates in the stools of patients with colorectal tumors (Chirouze et al., 2013; Kaindi et al., 2018). In 2005, Vanhoutte et al. (2005) conducted a study on the stability of the gut microbiome after administration of prebiotics, and recommended investigation of *S. equinus-infantarius-lutetiensis* ecology and its role in the gut of healthy dogs, since it was the streptococcal group with the most pronounced population growth observed after administration of the prebiotic, fructan. *S. equinus-infantarius-lutetiensis* was also isolated in a cat with intestinal lymphoma (Piva et al., 2019) and in the equine hindgut, in conjunction with oligofructose-induced laminitis (Milinovich et al., 2008). All of these studies point towards *S. equinus-infantarius-lutetiensis* being a pathobiont, i.e., a commensal bacteria normally present in the gut of healthy humans and other animals, with the potential to either cause serious infections or activate the immune system, causing inflammatory diseases (Chow et al., 2011; Jans and Boleij, 2018). Thus, by being more permissive of this strain, the Lundehund genetic background may increase the risk of *S. equinus-infantarius-lutetiensis* causing pathology.

The fact that the principal component analysis performed on a subset of purebred Lundehunds did not show any difference in fecal microbiome composition between the healthy individuals and that with a history of Lundehund syndrome might indicate that all purebred Lundehunds are genetically predisposed to dysbiosis.

By comparing the microbiome of Lundehunds with that of first and second-generation outcrossings with Buhund, we concluded that Lundehunds have a highly varied microbiome and that Lundehund syndrome is characterized by a dysbiotic state, similar to what is observed in humans and dogs with IBD, which is consistent with the disease etiology. However, the F/B ratio was higher in Lundehunds which have had Lundehund syndrome, whereas a lower F/B ratio is observed in humans and dogs with Crohn’s disease (an IBD type) (Minamoto et al., 2015), a difference that might be affected by many factors including both genetic and environmental differences between dogs and humans.

We propose that the loss of microbiome consistency in purebred Lundehunds is due to the loss of genetic loci that are needed for microbiome colonization stability. We further propose that these genetic loci were regained by outcrossing with Buhund in the F1 and F2 dogs, leading to a more stable gut microbiome. Further studies linking microbiome traits with genetic markers at individual level will help elucidate the mechanisms behind gut microbiome specificity in vertebrates.

## Data availability statement

The data presented in this study are available on Dryad, at the URL: https://datadryad.org/stash/dataset/doi:10.5061/dryad.tb2rbp05m.

## Ethics statement

Ethical review and approval were not required for this study because we only collected stool samples and this non-invasive procedure does not require evaluation by an ethics committee in Norway. The owners of the animals were provided with written information about the study and agreed to participate to it by sending stool samples of their dogs.

## Author contributions

CM and WL contributed to conception and design of the study and wrote the first draft of the manuscript. CM and P-AW did the data collection. CM and AB performed the statistical analyses. All authors contributed to manuscript revision and approved the submitted version.

## Funding

This study was supported by Queen Maud University College, the Norwegian Agriculture Agency (grant Agros 1135359), the Peder Sather Center for Advanced Study, and the Norwegian Lundehund Club. WL was supported by NIH DP5OD017851, NSF IOS 2144342, and the Carnegie Institution for Science endowment.

## Conflict of interest

The authors declare that the research was conducted in the absence of any commercial or financial relationships that could be construed as a potential conflict of interest.

## Publisher’s note

All claims expressed in this article are solely those of the authors and do not necessarily represent those of their affiliated organizations, or those of the publisher, the editors and the reviewers. Any product that may be evaluated in this article, or claim that may be made by its manufacturer, is not guaranteed or endorsed by the publisher.
